# Draft genome sequencing data of *Enterococcus faecium* BT22, a vancomycin-resistant opportunistic pathogen isolated from hospital effluents

**DOI:** 10.1016/j.dib.2023.109664

**Published:** 2023-10-11

**Authors:** Taha Ahmed Benabbou, Lotfi Ghellai, Mokhtar Benreguieg, Yahia Bellil, Meriem Mokhtar, Wassila Chahrour

**Affiliations:** a Department of Biology, Faculty of Sciences, University of Saida Dr Moulay Tahar, BP 138 ENNASR 20000, Saida, Algeria; b Laboratory of Biotoxicology, Pharmacognosy and Biological Valorisation of Plants, Compus Ain El Hadjar, Department of Biology, Faculty of Sciences, University of Saida Dr Moulay Tahar, BP 138 ENNASR 20000, Saida, Algeria; c Laboratory of microorganism's biology and biotechnology, University of Oran1 Ahmed Benbella, B.P. 16, Es‑Sénia, 31100 Oran, Algeria; d Laboratory of Applied Microbiology, Department of Biology, Faculty of Nature Science and Life, University of Oran 1Ahmed BENBELLA, B.P. 16, Es-Sénia, 31100 Oran, Algeria; e Laboratory of Bioeconomy, Food Safety and Health, Faculty of Natural Sciences and Life, University of Abdelhamid Ibn Badis, 27000 Mostaganem, Algeria

**Keywords:** Enterococcus faecium, Draft genome, Hospital effluents, Vancomycin-resistant enterococci

## Abstract

A vancomycin-resistant Gram-positive bacterium of the genus *Enterococcus*, designated as BT22, was isolated from untreated hospital effluents at Chettia Chlef Hospital. The complete genome of strain BT22 was sequenced using the Illumina MiSeq platform, revealing a total length of 2,577,707 bp, with 2462 coding sequences (CDS) and an average G+C content of 38.00 mol%. Phylogenomic analyses confirmed that strain BT22 belongs to the same species as *Enterococcus faecium* AVS0243, with a similarity of 99.79 %. The study identified 12 antibiotic resistance genes and one virulence gene in strain BT22. These genes confer resistance to various classes of antibiotics, including aminoglycosides, macrolides, tetracyclines, and vancomycin. However, the virulence gene identified codes for adhesion. Furthermore, mobile genetic elements, such as IS elements carried by a conjugative plasmid, were detected. The genomic sequencing data of *E. faecium* BT22 will be of great value to the scientific community, enabling comparative genomic analyses and a better understanding of antibiotic resistance mechanisms, particularly towards vancomycin. The genomic information has been deposited in the DDBJ/EMBL/GenBank databases under accession number JASSVD010000000, providing an essential resource in the fight against antibiotic resistance and the spread of resistant bacterial strains.

Specifications Table

**Table:** 

Subject	*Biology*
Specific subject area *Microbiology, genomics*	Specific subject area *Microbiology, genomics*
Type of data	Table Figure Draft genome sequence
How the data were acquired	Genome sequencing was performed on Illumina MiSeq Sequencer at Microsynth AG, Schützenstrasse 15, 9436 Balgach, Switzerland. The essential software tools used include Trimmomatic for read cleaning, MIRA for assembly, Pilon for consensus improvement, CONTIGuator for contig order, and Quast for assembly quality evaluation. Annotation was carried out with PGAP, and phylogenetic analysis with OAT. The search for antibiotic resistance and virulence genes was performed using ABRicate. Additionally, a circular map was generated using Proksee. Finally, the detection of plasmids, prophages, and the mobilome was conducted with PlasmidFinder 2.0, PHASTER, and VRprofile2, respectively, while OriTfinder was used to identify sites of mobile genetic element transfer origin.
Data format	Raw, analyzed and assembled genome sequence
Description of data collection	A pure culture of *Enterococcus faecium* BT22 was routinely cultured in BHI medium at 37°C. Genomic DNA was extracted from a 24-hour culture on BHI and used as a template for the sequencing reaction.
Data source location	*Institution: University of Saida Dr Moulay Tahar• City/Town/Region: Saida ENNASR • Country: Algeria • Latitude and longitude:* 34.859 N 0.152 E
Data accessibility	The complete genome sequence of *Enterococcus faecium* BT22 was deposited in NCBI GenBank under accession number JASSVD010000000 Direct URL to data: https://www.ncbi.nlm.nih.gov/bioproject/979350 Database link: BioProject: PRJNA979350, BioSample: SAMN35578370, SRA: SRR26034093

## 1 Value of the Data

• Preliminary genomic data of *Enterococcus faecium* strain BT22, isolated from untreated hospital effluents, provide a valuable opportunity to deepen our understanding of antibiotic resistance mechanisms, especially vancomycin resistance, developed in a hospital setting.
• These data are crucial, essential for exploring the virulence of BT22 and the mechanisms of acquisition and horizontal transfer of resistance, thus offering perspectives on combating infections.
• These data hold significant importance for obtaining information on antimicrobial resistance genes in vancomycin-resistant *Enterococcus faecium* strains, benefiting researchers and the fields of medicine and health.
• By unraveling the genome of this strain, these data can serve as a reference sequence for detecting antibiotic resistance genes in other isolates with unknown genomic information.

## 2 Objectives

The primary objective of this study is to provide the complete genome sequence of *E. faecium* strain BT22, thereby facilitating an in-depth exploration of genes associated with vancomycin resistance, as well as the identification of virulence genes and those involved in horizontal transfer.

## 3 Data Description

**Fig. 1:**
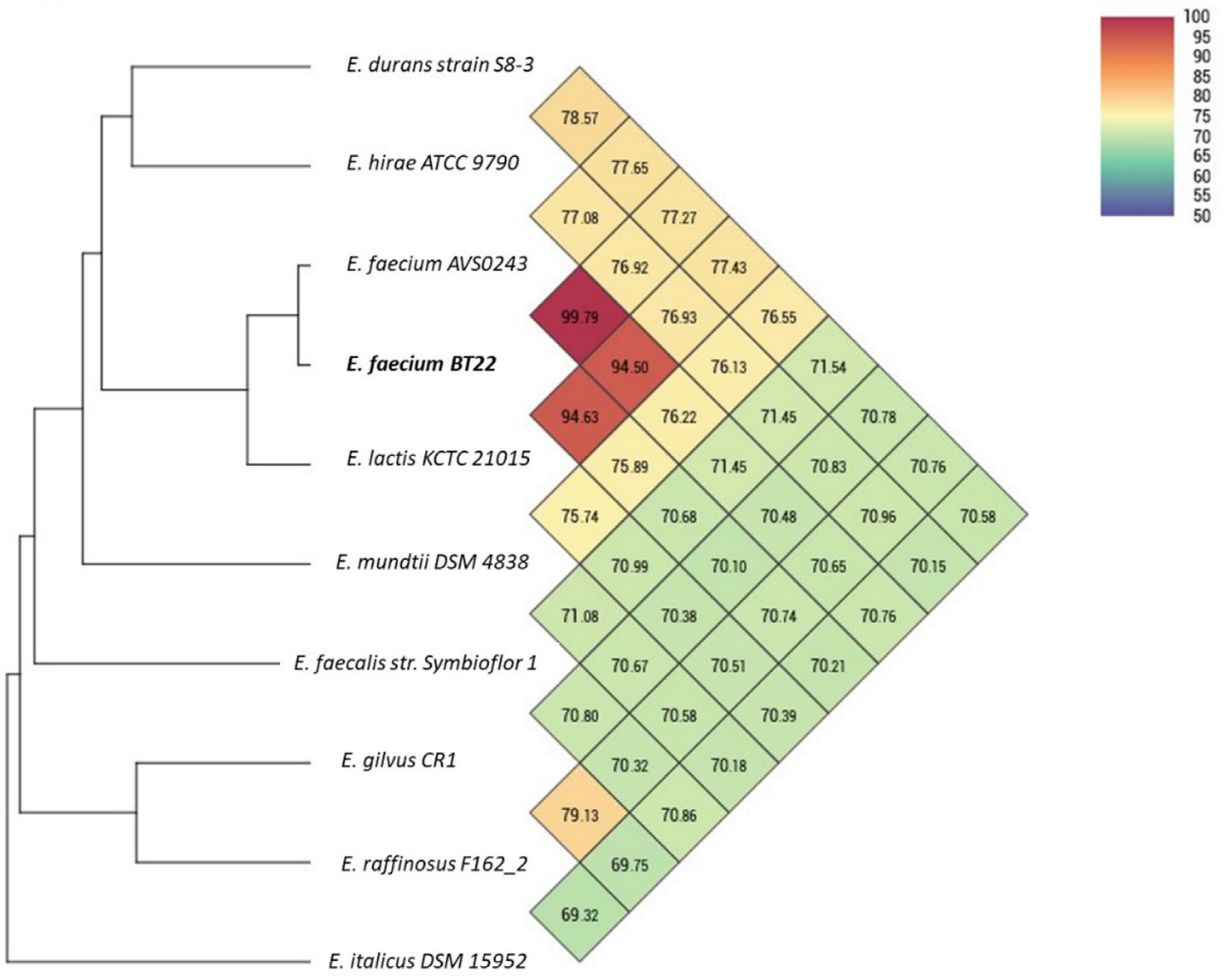
Heat Map and Phylogenetic Tree of Average Nucleotide Identity (ANI) for BT22 Genome and Closely Related *Enterococcus* spp. The heat map on the right is shown as an oblique 10 × 10 matrix, where each cell represents the ANI value between the row and the corresponding genome of the column, The color keys represent the identity of strains with lower (blue), and higher (red) ANI values and the phylogenetic tree on the left is a distance tree constructed by the UPGMA method.

The strain BT22 originates from untreated hospital effluent samples collected at Chettia Chlef Hospital. This strain has been identified as Vancomycin-Resistant *Enterococcus* (VRE), with a minimum inhibitory concentration (MIC) reaching up to 1024 µg/mL. Based on the complete genome sequence, isolate BT22 was closely related to *Enterococcus faecium* AVS0243 with a similarity of 99.79 %. The phylogenomic tree also confirmed that strain BT22 belongs to *E. faecium* (Fig. 1). The preliminary genome consists of 91 contigs, with a total length of 2,577,707 base pairs (bp), an N50 value of 65,181, and an L50 value of 14. In this genome, there are 2462 coding sequences (CDS), 43 tRNA genes, and 4 rRNA genes, with a G+C content of 38.00 mol%. All this genomic data has been deposited in GenBank and is accessible at the following address: https://www.ncbi.nlm.nih.gov/bioproject/979350.

We have successfully identified 12 antibiotic resistance genes and one virulence gene in strain BT22 (Table 1). Among these genes, *aac(6′)-Ii* and str confer resistance to aminoglycosides, while *msrC, tet(M),* and *eatA* are responsible for resistance to macrolides, tetracyclines, and LSAP (Lincosamides, Streptogramins A, and Pleuromutilins), respectively. Additionally, we identified seven other genes associated with vancomycin resistance, such as *vanZ-A, vanY-A, vanX-A, vanA, vanH-A, vanS-A, and vanR-A*. We also detected a virulence gene “*acm*” that plays an important role in the adhesion of *Enterococcus* bacteria.

**Table 1:** Antibiotic resistance genes and virulence factors found in BT22 genome.

	Categories	Genes	%cov	%Id	Contig	Product	Accession of closest sequence
AMR	Macrolide	*msr(C)*	100	98.92	3	ABC-F type ribosomal protection protein *Msr(C)*	WP_063854349.1
	Aminoglycoside	*aac(6′)-Ii*	100	99.82	10	Aminoglycoside N-acetyltransferase *AAC(6′)-Ii*	WP_002293989.1
	LSAP	*eat(A)*	100	99.27	15	ABC-F type ribosomal protection protein *Eat(A)*	WP_002296175.1
	Tetracycline	*tet (M)*	100	100	35	Tetracycline resistance ribosomal protection protein *Tet(M)*	WP_000691749.1
	Streptomycin	*str*	66.67	93.02	36	Streptomycin adenylyltransferase *Str*	WP_001258486.1
	Vancomycin	*vanZ-A*	100	100	36	Glycopeptide resistance protein *VanZ-A*	WP_000516404.1
		*vanY-A*	100	100	36	D-Ala-D-Ala carboxypeptidase *VanY-A*	WP_001812592.1
		*vanX-A*	100	100	36	D-Ala-D-Ala dipeptidase *VanX-A*	WP_000402347.1
		*vanA*	100	100	36	D-alanine–(R)-lactate ligase *VanA*	WP_001079845.1
		*vanH-A*	100	100	36	D-lactate dehydrogenase *VanH-A*	WP_001059542.1
		*vanS-A*	100	100	36	VanA-type vancomycin resistance histidine kinase *VanS*	WP_002305818.1
		*vanR-A*	100	100	36	Vancomycin resistance response regulator transcription factor *VanR-A*	WP_001280781.1
VF	Adherence	*acm*	86.1	99.46	10	(*acm*) collagen adhesin precursor *Acm*	AAN12397

The genome of BT22 revealed three sequences showing strong homology with plasmid sequences, as identified using PlasmidFinder 2.0 (Table 2). Additionally, during the search for prophages using PHAST, we identified an intact prophage region within contig 18, located between positions 420-40866. This prophage region contains all the necessary elements for a functional phage, including the *attL* and *attR* attachment sites of the phage, the integrase, tail, head, capsid, portal, and genes encoding the terminase.

**Table 2:** Plasmid Sequences Identified in the Genome of *E. faecium* BT22.

Plasmid	Identity	Length	Contig	Position	Note	Accession number
repUS43	99,83	1206/1206	Contig 35	18468-19673	CDS12738 (DOp1)	CP003584
rep1	100	1491/1491	Contig 36	2120-3610	repE (pIP816)	AM932524
rep2	100	1494/1494	Contig 68	2030-3523	orf1 (pRE25)	X92945

VRprofile2 allowed us to highlight the presence of mobile genetic elements, such as Tn/IS, some of which were carried by plasmid replicons (Table 3). These elements could be involved in the mobilization of resistance genes, particularly IS1297, which has been associated with the transfer of the str resistance gene related to Streptomycin, as well as the *VanHAX* genes related to Vancomycin and Teicoplanin resistance. An additional analysis of the replicon containing IS1297, the rep1 plasmid, conducted with the help of OriTfinder, revealed that it is conjugative. This plasmid possesses all the necessary genes for conjugation, including the relaxase, *oriT, T4SS* gene cluster, and a *T4CP* coupling protein.

**Table 3:** Mobilome associated with antibiotic resistance in the Genome of *E. faecium* BT22.

Contig	Replicon	Details	Position	Length	Genes	Drugs
Contig_2	Chromosome	ISAac3 / ISEfa7	123326-126816	3490	–	–
Contig_20	Chromosome	ISEfa10	15785-16486	701	–	–
Contig_36	Plasmid	IS1297	8356-14493	6137	*str / VanHAX*	Streptomycin, Vancomycin,Teicoplanin
Contig_36	Plasmid	ISLmo3	21114-21476	362	–	–
Contig_47	Plasmid	ISEfa8 / ISEfa8 / IS1476	253-2393	2140	–	–
Contig_72	Uncertain	IS1252 / IS1062 / IS1062	151-3764	3613	–	–

**Fig. 2:**
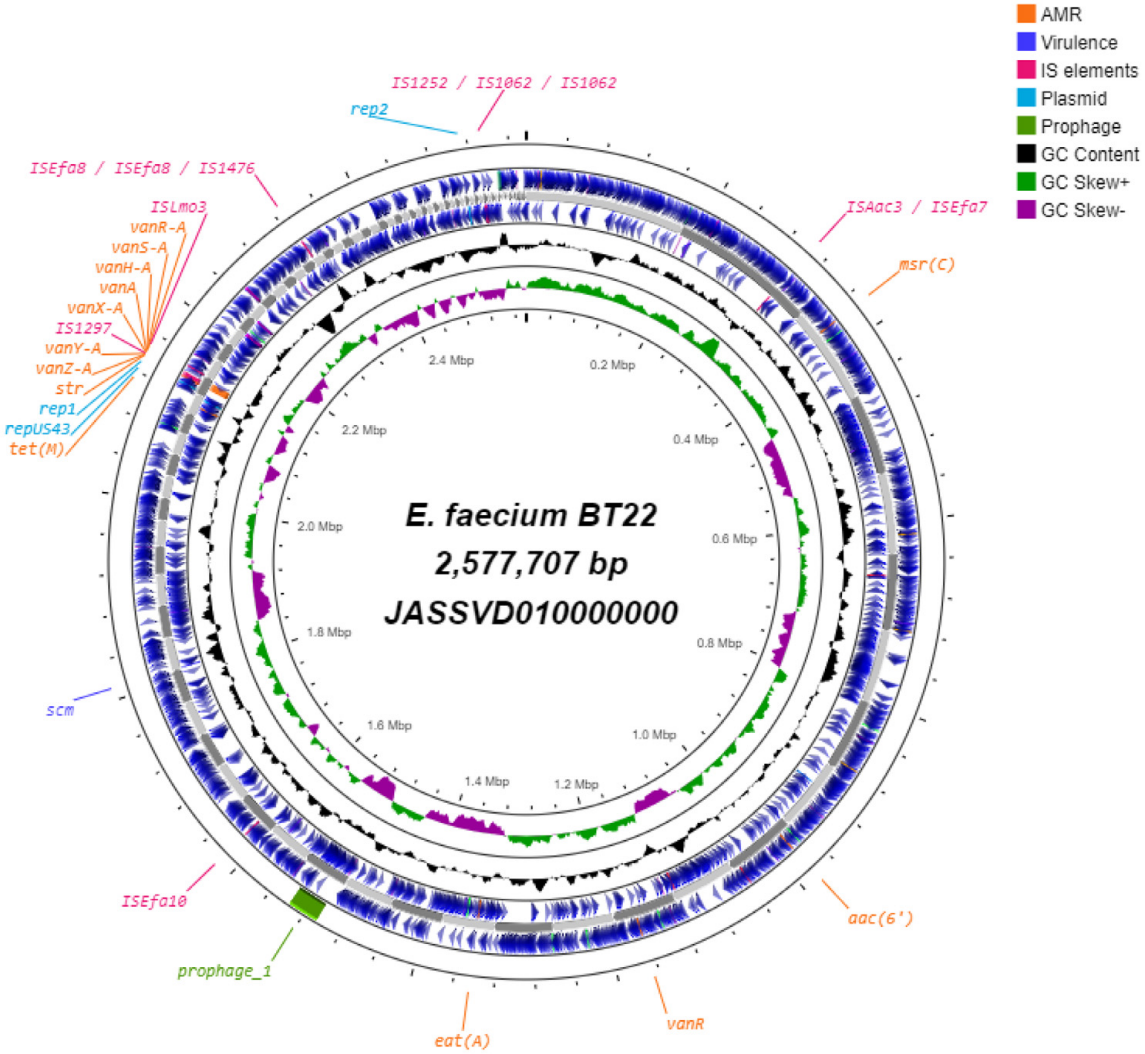
Circular Genome Map of *E. faecium* BT22 (JASSVD010000000): Tracks from the inside out include GC skew, GC content, contigs, forward genes, reverse genes, antibiotic resistance genes, virulence genes, IS elements, plasmid regions, and prophage regions. This circular genomic map was constructed using Proksee.

The circular genome map of *E. faecium* BT22 shows the genome distribution (Fig. 2)

## 4 Experimental Design, Materials and Methods

### 4.1 Processing of raw sequences and assembly, annotation, and analysis

Strain BT22 was isolated from untreated hospital effluent samples collected at Chettia Chlef Hospital. It was obtained using a dilution method on Slanetz and Bartley agar plates containing vancomycin at a concentration of 8 µg/mL. Identification was performed through experiments involving colony morphology, physiological and biochemical experiments, as well as MALDI-TOF identification. After obtaining a pure colony of strain BT22, we conducted a subculture in 2 mL of BHI broth medium, followed by incubation at 37°C for 18 hours. Subsequently, we extracted the total genomic DNA following the manufacturer's instructions of the DNAMITE DNA bacterial kit from Microzone Ltd (United Kingdom). Before proceeding further, we verified the concentration of the purified DNA using Qubit (Qubit 2.0 Fluorometer) and Nanodrop (NanoDrop 2000) measurement devices. For the subsequent analysis, we sent the purified DNA sample to the service provider Microsynth AG, Schützenstrasse 15, 9436 Balgach, Switzerland. Their expertise in genomic sequencing was utilized to perform the sequencing on the Illumina MiSeq platform. To prepare the DNA library for sequencing, we used the Nextera XT DNA Library Prep 2 × 300 bp v3 reagent kits provided by Illumina (San Diego, CA).

### 4.2 Whole genome sequencing, assembly annotation and analysis

The raw sequences underwent quality checking using FASTQC [1]. Poor-quality reads and adapters were removed using Trimmomatic [2], leaving only filtered reads for assembly with MIRA v4.9.6 [3]. The consensus was improved by aligning Illumina reads to the contigs using Pilon [4]. CONTIGuator [5] was used to determine the order and orientation of the contigs, while Quast 5.0.2 [6] was utilized to examine and statistically assess the assembly quality. The predicted genes were used as the input for BUSCO [7] to estimate genome completeness based on the lactobacillales_odb10 dataset.

Genome annotation was performed using PGAP from NCBI [8], and OAT [9] was employed to establish phylogenetic relationships and genome similarity, resulting in the construction of a phylogenetic tree and a heatmap. The search for antibiotic resistance genes and virulence genes in strain BT22 was conducted in the AMRFinderPlus [10] and VFDB [11] databases using ABRicate [12]. The search for plasmids, prophages, and detection of the mobilome associated with antibiotic resistance were conducted using PlasmidFinder 2.0 [13], PHASTER [14], and VRprofile2 [15], respectively. Furthermore, OriTfinder [16] was employed to rapidly locate the origin sites for DNA transfer in mobile genetic elements. Finally, a circular genomic map was generated using Proksee [17]

## Ethics Statements

This study did not involve any human subjects and animal experiments. No ethical approval was required.

## CRediT authorship contribution statement

**Taha Ahmed Benabbou:** Supervision, Software, Data curation. **Lotfi Ghellai:** Investigation, Formal analysis. **Mokhtar Benreguieg:** Methodology, Conceptualization, Validation. **Yahia Bellil:** Methodology. **Meriem Mokhtar:** . **Wassila Chahrour:** Methodology.

## Data Availability

DDBJ/EMBL/GenBank (Original data) (DDBJ/EMBL/GenBank databases).
